# Activating Cannabinoid Receptor 2 Protects Against Diabetic Cardiomyopathy Through Autophagy Induction

**DOI:** 10.3389/fphar.2018.01292

**Published:** 2018-11-06

**Authors:** Aiping Wu, Pengfei Hu, Jian Lin, Wan Xia, Rui Zhang

**Affiliations:** ^1^Department of Rehabilitation Medicine, Zhejiang Hospital, Hangzhou, China; ^2^Department of Cardiology, The Second Affiliated Hospital of Zhejiang Chinese Medical University, Hangzhou, China

**Keywords:** cannabinoid receptor 2, diabetic cardiomyopathy, autophagy, cardiomyocyte, high glucose

## Abstract

Cannabinoid receptor 2 (CB_2_) has been reported to produce a cardio-protective effect in cardiovascular diseases such as myocardial infarction. Here in this study, we investigated the role of CB_2_ in diabetic cardiomyopathy (DCM) and its underlying mechanisms. HU308 was used for the selective activation of CB_2_. Bafilomycin A1 was used for the blockade of autophagy and compound C was used to inhibit AMPK signaling. An streptozotocin (STZ)-induced mice model and high glucose (HG)-challenged cardiomyocytes were applied for study. Cardiac function was detected by echocardiography and Western blot for the detection of autophagy-related and its signaling-related proteins. Transmission electron microscopy was used for the analysis of autophagosome number. Cell viability was detected by Cell Counting Kit-8 (CCK-8) and lactate dehydrogenase (LDH) release assays. We found that activating CB_2_ by HU308 improved cardiac function in DCM as well as cell viability in cardiomyocytes under HG challenge, while the administration of bafilomycin A1 attenuated the protective effects. HU308 enhanced the level of autophagy in the heart tissues from DCM mice as well as cardiomyocytes under HG challenge. HU308 triggered the AMPK-mTOR-p70S6K signaling pathway, while the administration of compound C attenuated the cardio-protective effect of HU308 in cardiomyocytes under HG challenge. In conclusion, we initially demonstrated that activating CB_2_ produced a cardio-protective effect in DCM as well as cardiomyocytes under HG challenge through inducing the AMPK-mTOR-p70S6K signaling-mediated autophagy.

## Introduction

Diabetic cardiomyopathy (DCM) refers to a disorder of the heart muscle in people with diabetes in the absence of coronary artery disease or hypertension, serving as a major cause of heart failure in diabetic patients (Picano, 2003; Avogaro et al., 2004; Wang et al., 2016). Since diabetic patients have been reported to have higher risk than those without diabetes in heart failure (twofold higher in male and fivefold higher in female), it is urgent to gain knowledge and manage the pathogenesis and progression of DCM (Preis et al., 2009; Xiao et al., 2018). So far, several mechanisms have been demonstrated to be involved in the onset of DCM, including disturbance of cardiac metabolism, over-activated inflammatory response and oxidative stress, thus contributing to the cell death of cardiomyocytes (Palomer et al., 2018; Pei et al., 2018; Varma et al., 2018; Xin et al., 2018). However, the specific mechanism of DCM pathogenesis is complicated and more effective therapies are demanded.

Cannabinoid receptor 2 (CB_2_), together with CB_1_, are members of cannabinoid receptors which belong to G protein-coupled receptor superfamily (Zou and Kumar, 2018). Unlike CB_1_ which is mainly distributed in the central nervous system, CB_2_ is widely spread in peripheral tissues and cells in the immune system like macrophages and T cells as well as other peripheral tissues like the heart tissue (Li et al., 2016; Han et al., 2017). Activating CB_2_ has been reported to produce a cardio-protective effect in myocardial ischemia as well as other cardiovascular diseases like atherosclerosis (Moris et al., 2015; Maslov et al., 2016; Maslov and Karpov, 2017). However, whether activating CB_2_ protects against DCM has not been elucidated.

Autophagy is a self-degradative and recycling process relying on lysosomes. It is a vital metabolic process targeting on long-lived protein and dysfunctional organelles (Shao et al., 2016; Cortes and La Spada, 2018). Autophagy has been reported to be involved in the regulation of various kinds of cardiovascular diseases, such as myocardial infarction and atherosclerosis (Kim and Lee, 2014; Saha et al., 2018). In DCM, it was demonstrated that increasing autophagy process protected against DCM (Wang et al., 2017; Yang et al., 2017). As a result, pharmacological induction of autophagy might provide a potential pathway for the treatment of DCM. Autophagy is also involved in the functions of numerous receptors. For the relations between autophagy and CB_2_, it has been demonstrated that autophagy is associated with the protective functions of CB_2_ in several kinds of diseases (Shao et al., 2014; Denaes et al., 2016; Ke et al., 2016), indicating the potential value of autophagy in the treatment of diseases.

Hence, we raised the hypothesis that activating CB_2_ produced a cardio-protective effect in DCM as well as cardiomyocytes under HG challenge through the induction of cardiac autophagy. We aimed to investigate the role of CB_2_ in the pathogenesis of DCM as well as analyzing whether autophagy in cardiomyocytes was involved in this process.

## Materials and Methods

### Animal Care and Use

C57BL/6J mice (8–10 weeks old, male) were purchased from Shanghai Super-B&K Laboratory Animal Corp. Ltd. (Shanghai, China). Mice had free access to water and standard chow diet (Shanghai Super-B&K Laboratory Animal Corp. Ltd., Shanghai, China). Experimental mice were housed in specific-pathogen-free conditions under a 12/12 h light cycle (on: 8:00 am) at 23 ± 2°C and 60 ± 10% humidity. All animal experiments were approved and conducted in accordance with the guidelines of Zhejiang Hospital.

### Creation and Treatment of DCM Mice

Diabetic cardiomyopathy mice model was created by intraperitoneal injection of streptozotocin (STZ) (dissolved in 0.1 mol/L citrate buffer, pH 4.5) at the dose of 50 mg/kg body weight per day for five consecutive days as previously described (Zhang et al., 2017; Xiao et al., 2018). Citrate buffer without STZ loading was injected to mice in an equal volume as control. One week after the final STZ injection, fasting blood glucose was consecutively detected twice and the mean value was calculated. Only those with mean value of fasting blood glucose more than 16.6 mmol/L were considered as diabetic mice. For certain groups, mice were treated with HU308 (3 mg/kg body weight, TOCRIS Bioscience, Bristol, United Kingdom), a specific agonist for CB_2_ (Hanus et al., 1999), and/or bafilomycin A1 (0.3 mg/kg body weight, Selleckchem, Houston, TX, United States) every day from 12 to 14 weeks after the final injection of STZ. Normal saline in same volume was injected as vehicle.

### Echocardiographic Examination

After the final injection of STZ for 14 weeks, mice were anesthetized with isoflurane (5%) using a ventilation equipment and subjected to a 2-D guided M-mode echocardiography in a Vevo 2100 system (Vevo 2100, Visual Sonics) for echocardiographic examination as previously described (Cen et al., 2017). The measurement of ejection fraction (EF) (%), fractional shortening (FS) (%), left ventricular end-systolic diameter (LVESD) and left ventricular end-diastolic diameter (LVEDD) were based on the analysis of at least 10 separate cardiac cycles. The equations of those measurements for calculation were listed as follows: LVESD = 7.0 × LVESD^3^/(2.4 + LVESD); LVESD = 7.0 × LVEDD^3^/(2.4 + LVEDD); EF (%) = (LVEDV – LVESV)/LVESV × 100%; EF (%) = (LVEDD - LVESD)/LVESD × 100%.

### Non-invasive Blood Pressure Measurement

After the final injection of STZ for 14 weeks, systolic blood pressure (SBP) and diastolic blood pressure (DBP) were measured by tail-cuff manometry using a CODATM non-invasive monitoring system as previously described (Wang et al., 2018). In brief, non-anesthetized and warmed mice were trained 3 days in a restrainer for tail-cuff inflation. Recording cuffs were then placed over the tail and the blood pressure was measured for 10 acclimation cycles followed by 20 measurement cycles.

### Culture and Treatment of Murine Ventricular Cardiomyocytes

For the culture of murine primary ventricular cardiomyocytes, the heart issues were harvested from neonatal C57BL/6J mice as described previously (Wu et al., 2016). In brief, heart issues obtained were dissociated with 0.05% trypsin (Gibco, Grand Island, NY, United States) and 0.08% type II collagenase (Gibco, United States). Cells were resuspended in Dulbecco’s modification of Eagle’s medium supplemented with 5.5 mM glucose (DMEM; Gibco) with 10% fetal bovine serum (FBS) and 0.1% penicillin–streptomycin and plated onto a 25-cm^2^ cell culture flask for 90 min at 37°C in a 5% CO_2_ incubator. Unadherent cells were plated onto 6-well (1 × 10^6^ cells/well) or 96-well (1 × 10^5^ cells/well) plates and cultured at 37°C in a 5% CO_2_ incubator. For *in vitro* studies, murine primary ventricular cardiomyocytes were cultured in medium with high glucose concentration (HG; 33 mM glucose) for 24 h as hyperglycemia group for the mimic of stimulation on the occurrence of DCM as described previously (Wei et al., 2018) or medium with normal glucose concentration (Nor; 5.5 mM glucose) as the control group. In certain groups, HU308 (10 μM) and/or bafilomycin A1 (5 nM) were treated to cells at 10 min before suffering HG. Compound C (10 μM, Sigma-Aldrich, St. Louis, MO, United States) was used for the inhibition of AMPK-mTOR-p70S6K signaling. PBS in same volume was administrated as vehicle.

### Cell Counting Kit-8 (CCK-8) Assay

A CCK-8 assay (Dojindo, Kamimashiki-gun Kumamoto, Japan) was used for the analysis of cell viability on murine ventricular cardiomyocytes according to the manufacturer’s protocol. In brief, murine ventricular cardiomyocytes were seeded in 96-well plates in the density of 1 × 10^5^ cells/well. After 24-h HG challenge, 10 μL CCK-8 reagent was added to each well for 4-h additional cultivation and the absorbance value was analyzed with a microplate reader (Tecan Group Ltd., Männedorf, Switzerland) at the wavelength of 450 nm.

### Lactate Dehydrogenase (LDH) Release

An lactate dehydrogenase (LDH) release assay (Dojindo, Kamimashiki-gun Kumamoto, Japan) was used for the detection of cell membrane integrity according to the manufacturer’s protocol. In brief, murine ventricular cardiomyocytes were seeded in 96-well plates in the density of 1 × 10^5^ cells/well. After 24-h HG challenge, LDH Working Solution was added to each well for the incubation of additional 30 min at 37°C. Then the Stop Solution was added and the absorbance value was analyzed with a microplate reader (Tecan Group Ltd., Switzerland) at the wavelength of 490 nm.

### Western Blot

Total proteins were extracted from heart issues or murine ventricular cardiomyocytes lysed in lysis buffer. Protein concentration was measured by Bicinchoninic acid method (Thermo Scientific, Pittsburgh, PA, United States). Samples were loaded in 6% or 15% Tris/Gly gels, and transferred on NC membranes through SDS-PAGE (Millipore, Billerica, MA, United States) followed by the incubation with primary and secondary antibodies. The primary antibodies applied were listed as follows: rabbit anti-Beclin-1 monoclonal antibody (1:1000; Cell Signaling Technology, Danvers, MA, United States), rabbit anti-LC3 polyclonal antibody (1:1000; Novus Biologicals, Littleton, CO, United States), rabbit anti-p62 antibody (1:1000; Cell Signaling Technology, Danvers, MA, United States), rabbit anti-adenosine 5′-monophosphate (AMP)-activated protein kinase (AMPK) antibody (1:1000, Cell Signaling Technology, Danvers, MA, United States), rabbit anti-phosphorylated AMPK antibody (1:1000, Cell Signaling Technology, Danvers, MA, United States) anti-mammalian target of rapamycin rabbit (mTOR) antibody (1:1000, Cell Signaling Technology, Danvers, MA, United States), rabbit anti-phosphorylated mTOR antibody (1:1000, Cell Signaling Technology, Danvers, MA, United States), rabbit anti-p70 ribosomal protein S6 kinase (p70S6K) antibody (1:1000, Cell Signaling Technology, Danvers, MA, United States), rabbit anti-phosphorylated p70S6K antibody (1:1000, Cell Signaling Technology, Danvers, MA, United States) and mouse anti-β-actin antibody (1:5000, Beyotime Biotechnology, Shanghai, China). The secondary antibodies applied were listed as follows: donkey anti-Rabbit and donkey anti-mouse secondary antibody (1:10000, LI-COR Biosciences, Lincoln, NE, United States). After incubation in antibodies, an Odyssey infrared imaging system (LI-COR Bioscience, Lincoln, NE, United States) was used for obtaining and analyzing images.

### Transmission Electron Microscopy

Murine ventricular cardiomyocytes were obtained and cultured at 37°C on 6-well plates followed by the treatments mentioned above. Cells were harvested and fixed overnight at 4°C in 2.5% glutaraldehyde in 0.1 M PBS, and then post-fixed in 1% buffered osmium tetroxide for 2 h. Samples were then processed in routine procedure and examined under a transmission electron microscope (H-700; Hitachi, Tokyo, Japan).

### Statistical Analysis

All data are presented as mean values ± SEM. Statistical analysis was conducted using one-way analysis of variance (ANOVA) followed by Bonferroni *post hoc* test for multiple comparisons, respectively. *P*-value < 0.05 was considered as statistical difference between groups.

## Results

### Activating CB_2_ Improves Cardiac Function in DCM Mice

**FIGURE 1:**
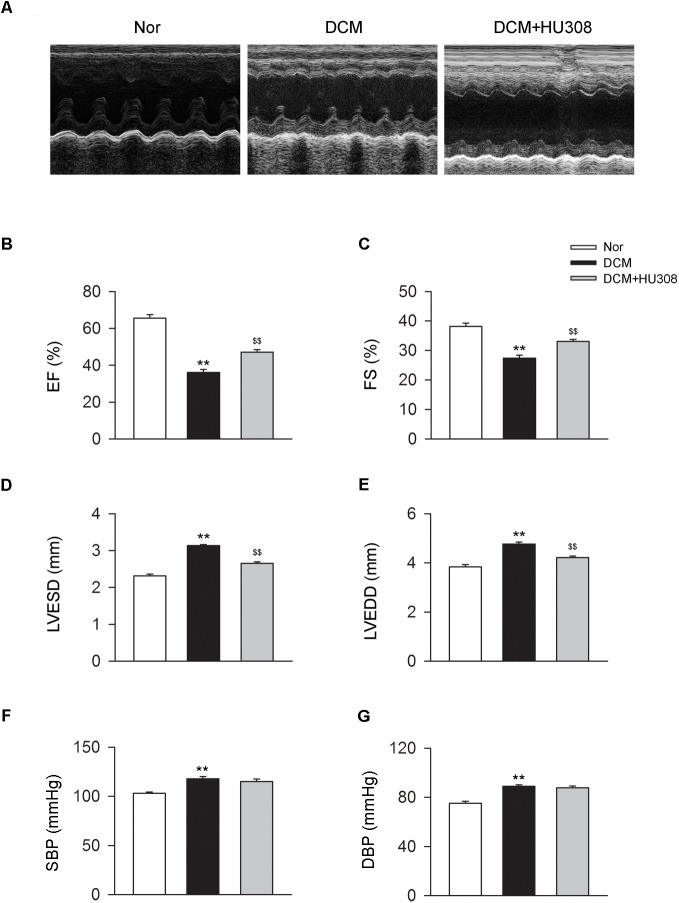
Administration of HU308 improves cardiac function in DCM mice. DCM mice model was created by intraperitoneal injection of streptozotocin (STZ) at the dose of 50 mg/kg body weight per day for five consecutive days. HU308 (3 mg/kg body weight) was administrated every day from 12 to 14 weeks after the final injection of STZ. Normal saline was used as vehicle. **(A)** Representative M-mode echocardiographic images on mice at 14 weeks after the final injection of STZ. **(B–E)** Quantitative analysis of cardiac function measurements including EF (%), FS (%), LVESD and LVEDD (*n* = 7). ^∗∗^*P* < 0.01 vs. Nor group; ^$$^*P* < 0.01 vs. DCM group. **(F,G)** Quantitative analysis of SBP and DBP (*n* = 7). ^∗∗^*P* < 0.01 vs. Nor group. Nor, normal; DCM, diabetic cardiomyopathy.

To assess the effect of activating CB_2_ on cardiac function in DCM mice, here we used HU308 for the selective activation of CB_2_ and conducted the echocardiographic examination for the analysis of cardiac function. We found that compared with the normal group, the occurrence of DCM deteriorated the damage of cardiac function in EF (%), FS (%), LVESD and LVEDD, while the administration of HU308 significantly attenuated those effects of DCM (Figures 1A–E). However, HU308 did not significantly affect the level of SBP or DBP in DCM mice models (Figures 1F,G). These data indicated the cardiac protective role of activating CB_2_ by HU308 on the occurrence of DCM.

### Activating CB_2_ Enhances Cardiac Autophagy in Heart Tissues From DCM Mice

**FIGURE 2:**
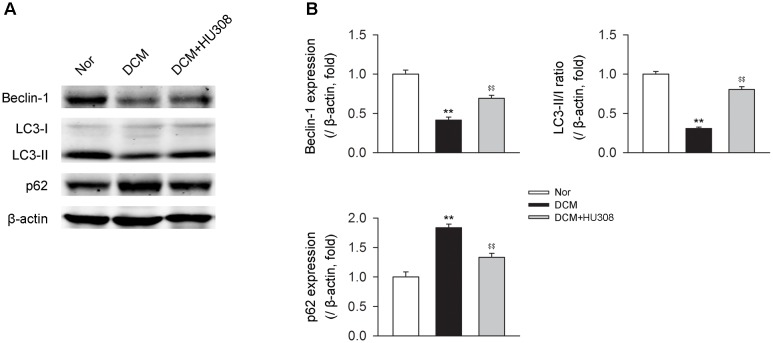
Administration of HU308 enhances cardiac autophagy in heart issues from DCM mice. **(A)** Representative images of the autophagy-related proteins including beclin-1, LC3-II/I, and p62. **(B)** Quantitative analysis of the autophagy-related proteins (*n* = 5). ^∗∗^*P* < 0.01 vs. Nor group; ^$$^*P* < 0.01 vs. DCM group. Nor, normal; DCM, diabetic cardiomyopathy.

Since autophagy has been reported to play an alleviative role in DCM, we then detected the effect of activating CB_2_ on autophagy process in heart issues on the occurrence of DCM. We found that compared with the normal group, heart tissues isolated from the DCM group showed a decrease in the levels of beclin-1 and LC3-II/I ratio while an increase in the level of p62. However, the administration of HU308 for the selective activation of CB_2_ significantly attenuated the decrease of beclin-1 and LC3-II/I ratio and increase of p62 compared with the DCM group (Figures 2A,B). Collectively, these data indicated that activating CB_2_ by HU308 enhanced the cardiac autophagy process in heart tissues on the occurrence of DCM.

### Blockade of Autophagy Deteriorates the Cardiac Protective Effect of Activating CB_2_ in DCM Mice

**FIGURE 3:**
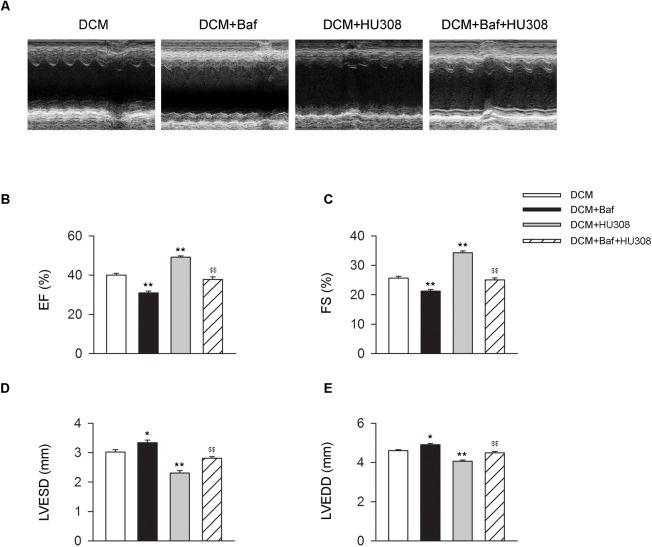
Bafilomycin A1 deteriorates the cardiac protective effect of HU308 in DCM mice. **(A)** Representative M-mode echocardiographic images on mice at 14 weeks after the final injection of STZ. **(B–E)** Quantitative analysis of cardiac function measurements including EF (%), FS (%), LVESD and LVEDD (*n* = 7). ^∗^*P* < 0.05 vs. DCM group; ^∗∗^*P* < 0.01 vs. DCM group; ^$$^*P* < 0.01 vs. DCM+HU308 group. DCM, diabetic cardiomyopathy; Baf, bafilomycin A1.

We then investigated whether HU308-induced autophagy was involved in the alleviation of cardiac function damage on the occurrence of DCM. Bafilomycin A1 was used for the inhibition of autophagy process. We found that the administration of HU308 for the activation of CB_2_ alleviated the damage of cardiac function compared with the DCM group, while bafilomycin A1 application significantly attenuated the protective effect of HU308 (Figures 3A–E). These data indicate that autophagy process was involved in the cardiac protective effect induced by activating HU308 on the occurrence of DCM.

### Activating CB_2_ Enhances Cardiac Autophagy in Cardiomyocytes Under the Challenge of HG

**FIGURE 4:**
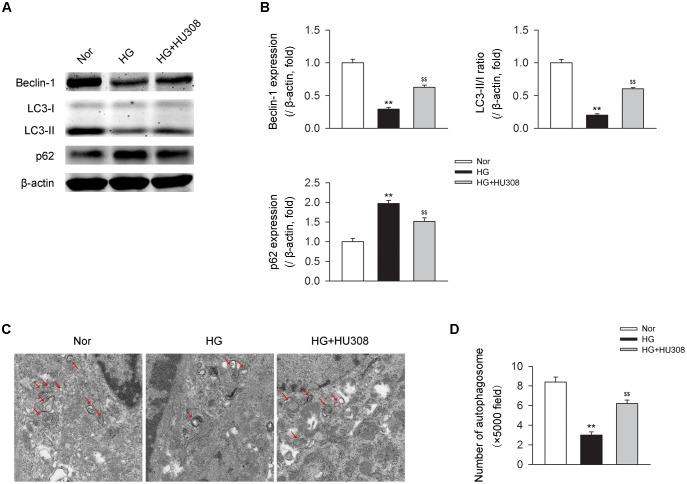
Administration of HU308 enhances cardiac autophagy in cardiomyocytes under the challenge of HG. Primary murine ventricular cardiomyocytes were obtained from neonatal C57BL/6J mice and challenged with high glucose concentration (HG; 33 mM glucose) for 24 h as hyperglycemia group or medium with normal glucose concentration (Nor; 5.5 mM glucose) as the control group. In certain groups, HU308 (10 μM) was treated to cells at 10 min before suffering HG. PBS in the same volume was administrated as vehicle. **(A)** Representative images of the autophagy-related proteins including beclin-1, LC3-II/I, and p62. **(B)** Quantitative analysis of the autophagy-related proteins (*n* = 5). ^∗∗^*P* < 0.01 vs. Nor group; ^$$^*P* < 0.01 *vs.* HG group. **(C)** Representative images of transmission electron microscopy with arrows marking autophagosomes. **(D)** Quantitative analysis of the number of autophagosomes in murine ventricular cardiomyocytes (*n* = 5). ^∗∗^*P* < 0.01 vs. Nor group; ^$$^*P* < 0.01 vs. HG group. Nor, normal; DCM, diabetic cardiomyopathy.

We then detected the effect of activating CB_2_ by HU308 on autophagy process in murine ventricular cardiomyocytes under HG challenge. We found that HG challenge decreased the level of autophagy through detecting the levels of autophagy-related proteins including beclin-1, LC3-II/I ratio and p62 (Figures 4A,B) and the number of autophagosomes (Figures 4C,D). However, the administration of HU308 significantly attenuated the decrease of autophagy led to by HG challenge. Taken together, these data indicated the increasing effect of activating CB_2_ on cardiac autophagy in cardiomyocytes under HG challenge.

### Activating CB_2_ Plays a Cardioprotective Role in Cardiomyocytes Under the Challenge of HG

**FIGURE 5:**
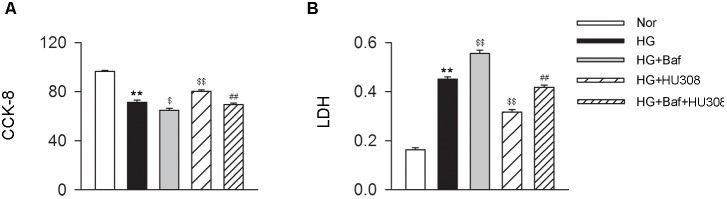
HU308-induced autophagy plays a cardio-protective role in cardiomyocytes under the challenge of HG. **(A)** Quantitative analysis of CCK-8 assay in murine ventricular cardiomyocytes (*n* = 6 per group). ^∗∗^*P* < 0.01 vs. Nor group; ^$^*P* < 0.05 vs. HG group; ^$$^*P* < 0.01 vs. HG group; ^##^*P* < 0.01 vs. HG+HU308 group. **(B)** Quantitative analysis of LDH release in murine ventricular cardiomyocytes (*n* = 6 per group). ^∗∗^*P* < 0.01 vs. Nor group; ^$$^*P* < 0.01 vs. HG group; ^##^*P* < 0.01 vs. HG+HU308 group. Nor, normal; DCM, diabetic cardiomyopathy; Baf, bafilomycin A1.

We further conducted the effects of CB_2_-induced autophagy on murine ventricular cardiomyocytes under HG challenge. CCK-8 and LDH assays were applied for the detection of cell viability. We found that the administration of HU308 for the activation of CB_2_ alleviated the damage of cell viability compared with the HG group, while bafilomycin A1 application significantly attenuated the protective effect of HU308 (Figures 5A,B). These data indicate that HU308-induced autophagy played a protective effect on murine ventricular cardiomyocytes under HG challenge.

### Participation of AMPK-mTOR-p70S6K Signaling in the Cardioprotective Effect of Activating CB_2_ in Cardiomyocytes Under the Challenge of HG

**FIGURE 6:**
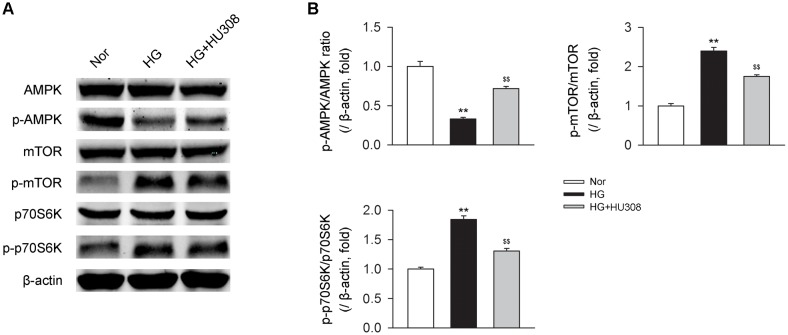
AMPK-mTOR-p70S6K signaling is triggered by HU308 in cardiomyocytes under the challenge of HG. **(A)** Representative images of the AMPK-mTOR-p70S6K signaling-related proteins. **(B)** Quantitative analysis of the relative p-AMPK/AMPK, p-mTOR/mTOR and p-p70S6K/p70S6K ratios (*n* = 5). ^∗∗^*P* < 0.01 vs. Nor group; ^$$^*P* < 0.01 vs. HG group. Nor, normal; DCM, diabetic cardiomyopathy.

**FIGURE 7:**
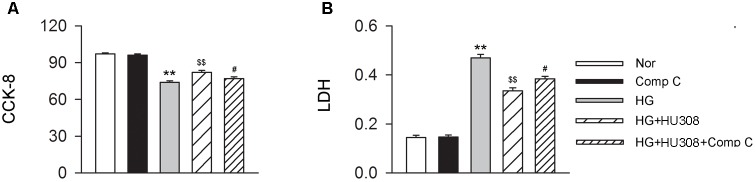
Participation of AMPK-mTOR-p70S6K axis in the cardioprotective effect of HU308 in cardiomyocytes under the challenge of HG. **(A)** Quantitative analysis of CCK-8 assay in murine ventricular cardiomyocytes (*n* = 6 per group). ^∗∗^*P* < 0.01 vs. Nor group; ^$$^*P* < 0.01 vs. HG group; ^#^*P* < 0.05 vs. HG+HU308 group. **(B)** Quantitative analysis of LDH release in murine ventricular cardiomyocytes (*n* = 6 per group). ^∗∗^*P* < 0.01 vs. Nor group; ^$$^*P* < 0.01 vs. HG group; ^#^*P* < 0.05 vs. HG+HU308 group. Nor, normal; DCM, diabetic cardiomyopathy; Comp C, compound C.

We finally investigated whether AMPK-mTOR-p70S6K signaling pathway, a classic autophagy signaling, was involved in the mediation of CB_2_-induced autophagy. We found that the administration of HU308 for the activation of CB_2_ significantly increased the level of p-AMPK/AMPK ratio and decreased the levels of p-mTOR/mTOR and p-p70S6K/p70S6K ratios compared with the HG group (Figures 6A,B). In addition, HU308 significantly attenuated the damage of cell viability led to by HG challenge, while pharmacological inhibition of the AMPK-mTOR-p70S6K signaling pathway by compound C attenuated the protective effect in cell viability (Figures 7A,B). Taken together, these data indicated the involvement of AMPK-mTOR-p70S6K signaling pathway in the cardio-protective effects of CB_2_-induced autophagy under HG challenge.

## Discussion

CB_2_ has been widely considered to be distributed mainly in peripheral tissues and cells including the immune system and heart tissue (Li et al., 2016; Han et al., 2017). It has been demonstrated that activating CB_2_ contributes to the suppression of inflammatory and immune responses, thus producing an alleviative effect on the pathogenesis and progression of various kinds of diseases (Steffens and Pacher, 2012; Espejo-Porras et al., 2018; Ratano et al., 2018). In the cardiovascular system, activating CB_2_ shows an attenuative effect on the pathogenesis of myocardial ischemia through the protection of cardiomyocytes and maintenance of cardiac function (Wang et al., 2014; Pacher et al., 2018). In other cardiovascular disorders such as atherosclerosis, activating CB_2_ was also reported to be protective through the inhibition of inflammatory reaction and lipid metabolism in plaque macrophages (Jiang et al., 2016; Maslov and Karpov, 2017). In addition, in diabetes, it was also demonstrated that deficiency of CB_2_ worsened the severity of diabetes as well as renal functional abnormalities in STZ-induced diabetic mice (Barutta et al., 2014). However, no evidence is provided to uncover the role of CB_2_ in DCM. Here in our current study, we demonstrated for the first time that selective activation of CB_2_ by HU308 significantly attenuated the damage of cardiac function through the detection of EF (%), FS (%), LVESD and LVEDD in STZ-induced DCM mice models. In addition, we further showed the cardio-protective effect of the administration of HU308 in cell viability in murine ventricular cardiomyocytes under HG challenge. Collectively, those data demonstrated the cardio-protective effect of activating CB_2_ in STZ-induced DCM mice models. We further provided evidence that HU308 did not significantly affect systemic blood pressure. Those results indicate that activating CB_2_ had primary effects on the heart without affecting systemic vasculature.

We then investigated the underlying mechanisms involving the CB_2_-mediated process. It was previously demonstrated that autophagy, a self-degradative process relying on lysosome, contributed to the alleviative effect mediated by activating CB_2_ in several inflammatory related diseases including multiple sclerosis, alcoholic liver disease and inflammatory bowel disease (Shao et al., 2014; Denaes et al., 2016; Ke et al., 2016). For example, Ke et al. (2016) demonstrated that activating CB_2_ alleviated inflammatory bowel disease in mice models via the inhibition of the NLRP3 inflammasome by inducing autophagy process in murine macrophages (Ke et al., 2016). Similar association between CB_2_ and autophagy was reported in mice multiple sclerosis models (Shao et al., 2014). For autophagy process in DCM, it was demonstrated that enhancing autophagy contributed to the amelioration of DCM (Wang et al., 2017; Yang et al., 2017; Pei et al., 2018; Xiao et al., 2018). So far, several agents have been reported to be effective in the attenuation of the pathogenesis and progression of DCM taking advantage of enhancing the level of autophagy process (Wang et al., 2017; Yang et al., 2017; Pei et al., 2018; Xiao et al., 2018). Here in this study, we initially reported that selective activation of CB_2_ via HU308 significantly enhanced the level of autophagy both in the heart tissues on the occurrence of DCM *in vivo* and cardiomyocytes under HG challenge *in vitro*. Furthermore, the administration of bafilomycin A1 to block autophagy process attenuated the cardio-protective effect of HU308 in the improvement of cardiac function in DCM mice models and cell viability in cardiomyocytes under HG challenge. Those data indicated that CB_2_-induced autophagy process was involved in the CB_2_-mediated cardio-protective effect.

We finally investigated the specific signaling mechanisms underlying. It was previously demonstrated that AMPK-mTOR-p70S6K signaling, a classical signaling of autophagy process, mediated the cardio-protective role of resveratrol, an autophagy inducer, in cardiomyocytes under HG challenge (Xu et al., 2018). In STZ-induced diabetic mice, AMPK-mTOR signaling contributed to the cardio-protective effect through the enhancement of autophagy process (Yang et al., 2017; Zhou et al., 2017). Consistent with those studies, in this study, we demonstrated that selective activation of CB_2_ by HU308 increased the phosphorylation of AMPK while decreasing the phosphorylation of mTOR and p70S6K, thus triggering the AMPK-mTOR-p70S6K signaling pathway in murine primary ventricular cardiomyocytes. Furthermore, the administration of compound C, an AMPK inhibitor, significantly attenuated the cardio-protective effect of HU308 through the detection of cell viability, indicating that AMPK-mTOR-p70S6K signaling-induced autophagy was involved in CB_2_-mediated cardiac protection in DCM. However, since the mechanisms on the CB_2_-mediated autophagy induction are complicated, further studies are demanded on this issue.

## Conclusion

Taken together, in this study, we initially showed that activating CB_2_ produced a cardio-protective effect in DCM as well as cardiomyocytes under HG challenge through the induction of the AMPK-mTOR-p70S6K signaling-mediated autophagy process. We believe that the findings of this study might enhance our knowledge on the understanding of the pathogenesis and progression of DCM and provide a novel insight in the development of therapeutic strategies against DCM.

## Ethics Statement

This study was carried out in accordance with the recommendations of the guidelines of the Animal Care Committee of Zhejiang Hospital, Zhejiang, China. The protocol was approved by the Animal Care Committee of Zhejiang Hospital.

## Author Contributions

AW and PH conducted all the experiments in animals and analyzed the data. JL conducted the experiments in cells. RZ designed the study and wrote the manuscript. WX revised the manuscript.

## Conflict of Interest Statement

The authors declare that the research was conducted in the absence of any commercial or financial relationships that could be construed as a potential conflict of interest.
